# Delipid extracorporeal lipoprotein filter from plasma system: a new intensive lipid lowering therapy for patients with acute ischemic stroke

**DOI:** 10.3389/fneur.2024.1342751

**Published:** 2024-03-06

**Authors:** Yuqiong Jiao, Qi Yang, Ting Ye, Jun Zhu, Qunyi Li, Xiang Han, Qiang Dong

**Affiliations:** ^1^Department of Neurology, Huashan Hospital, Fudan University, Shanghai, China; ^2^Department of Pharmacy, Huashan Hospital, Fudan University, Shanghai, China; ^3^National Clinical Research Center for Aging and Medicine, Huashan Hospital, Fudan University, Shanghai, China; ^4^State Key Laboratory of Medical Neurobiology, Fudan University, Shanghai, China

**Keywords:** low-density lipoprotein apheresis, delipid extracorporeal lipoprotein filter from plasma, intensive lipid lowering therapy, acute ischemic stroke, cerebral protection, low flow

## Abstract

**Objectives:**

To investigate the safety and efficacy of the delipid extracorporeal lipoprotein filter from plasma (DELP) system, a new low-density lipoprotein cholesterol (LDL-C) adsorption system, in acute ischemic stroke (AIS) patients.

**Patients and methods:**

In the present study, a total of 180 AIS patients were enrolled during March 2019 to February 2021. They were divided into DELP group (*n*_1_ = 90) and the control group (*n*_2_ = 90). The treatment protocol and vascular access of DELP treatment was established and evaluated. For the DELP group, clinical data and laboratory results including plasma lipid and safety parameters before and after the apheresis were collected and analyzed. For all participants, neurological scores were assessed and recorded.

**Results:**

For the DELP group, 90 patients including 70 males and 20 females were included. The mean LDL-C was significantly decreased from 3.15 ± 0.80 mmol/L to 2.18 ± 0.63 mmol/L (30.79%, *p* < 0.001) during a single DELP treatment, and decreased from 3.42 ± 0.87 mmol/L to 1.87 ± 0.48 mmol/L (45.32%, *p* < 0.001) after two DELP treatments. No clinically relevant changes were observed in hematologic safety parameters and blood pressure levels except for hematocrit and total protein throughout the whole period of DELP treatment. The DELP group showed improvement relative to the control group in National Institute of Health stroke scale scores (NIHSS) on the 14th and 90th day after stroke. Moreover, the DELP group had a significantly higher ratio of mRS 0 to 1 on the 90th day after stroke.

**Conclusion:**

The new LDL-C adsorption system, the DELP system, may provide a new option for intensive lipid lowering therapy in AIS patients in view of its safety, efficacy, and operation feasibility.

## Introduction

Intensive lipid lowering therapy after ischemic stroke is recommended by the current guideline of the American Heart Association and the American Stroke Association (AHA/ASA) (1–3). This recommendation is based on clinical trials which showed that lipid lowering therapy could reduce the recurrent risk of stroke (4, 5). Furthermore, evidence is accumulating that the more the low-density lipoprotein (LDL) cholesterol (LDL-C) was decreased, the lower the risk for ischemic events, without any threshold effects (6–8).

LDL-C lowering methods mainly include pharmacotherapy and extracorporeal lipoprotein apheresis. Compared with pharmacotherapy, lipoprotein apheresis may be more effective and provide a more rapid response (9). Dating back to the 1990s, heparin-mediated extracorporeal LDL precipitation (HELP), a type of lipoprotein apheresis, was reported to be safe and effective in lowering plasma lipid in ischemic stroke patients, and may obtain extra therapeutic success by providing immediate improvements in hemorheological situation and perfusion (10).

However, research on lipoprotein apheresis in acute ischemic stroke (AIS) has been limited. The clinical application and development of lipoprotxin apheresis in AIS may be limited by several factors. Firstly, a large volume of processed blood is needed in most lipoprotein apheresis systems, which may increase the risk of hypoperfusion and intolerance (10–12). Secondly, numerous publications have demonstrated the problem of vascular access as the major drawback to lipoprotein apheresis (9, 13). The vascular access plays an important role in patients’ tolerance and compliance to treatment, and the placement of peripheral access catheters and needles should be carefully considered. In addition, Doherty et al. reported that high cost, time commitments and strict requirements for practitioner expertise may also slow down the pace of investigation in lipoprotein apheresis (14).

In the present study, we introduced a new lipoprotein apheresis system, delipid extracorporeal lipoprotein filter from plasma (DELP) system based on JX-DELP depth delipid filter technology (Jiangxia Blood Technology Corporation, Shanghai, China). Compared with traditional lipoprotein apheresis systems, the DELP system may have advantages in some aspects, including ease of operation, reliable vascular access, and lower cost. We performed this study to provide the first data on the operating protocol, LDL-lowering capability and the safety of DELP system in patients with AIS.

## Patients and methods

### Patients

We assembled a retrospective cohort of patients with acute ischemic stroke at the Department of Neurology, Huashan Hospital, Fudan University from March 2019 to February 2021. Neurological scores data were extracted from the inpatient and outpatient electronic health record system. We enrolled patients who had a new diagnosis of ischemic stroke with plasma LDL-C level ≥ 2.0 mmol/L. Exclusion criteria: (1) patients who showed hypersensitivity to sodium citrate; (2) patients with severe arrhythmia; (3) patients with end-stage renal or hepatic failure; (4) patients with active infection; (5) Stroke previously or received statin treatment before; (6) Died or lose to follow up within 90 days.

All patients accepted guideline-recommended drug therapy for AIS. All patients were treated with atorvastatin (20 mg/day) for lipid regulation. Among the patients who did not receive thrombolytic thepapy, patients with minor stroke (NIHSS≤3) or predominantly intracranial symptomatic stenosis were treated with short-term dual antiplatelet (aspirin and clopidogrel) followed by long-term monoclonal antiplatelet. And the other patients were treated with monoclonal antiplatelet (aspirin 100 mg/day or clopidogrel 75 mg/day) (15, 16). Patients undergoing first DELP were treated within 7 days of symptom onset after procedural risks were explained in detail. Part of them may be treated with one more DELP treatment after 24 h if LDL-C did not reach the target level after a single DELP treatment.

From those patients with AIS, we selected patients with DELP and control (patients without DELP) by 1:1 pairwise matching. We indetified a set of control patients who were in the same age category (≤49, 50–59, 60–69, 70–79). We then randomly chose one control for each patient with DELP and matched them by age (Supplementary Figure 1). The study was approved by Ethics Committee of Huashan Hospital, Fudan University (approval number: 2017-336). Informed consents were obtained from all patients for DELP system therapy.

### DELP treatment protocol

**Figure 1:**
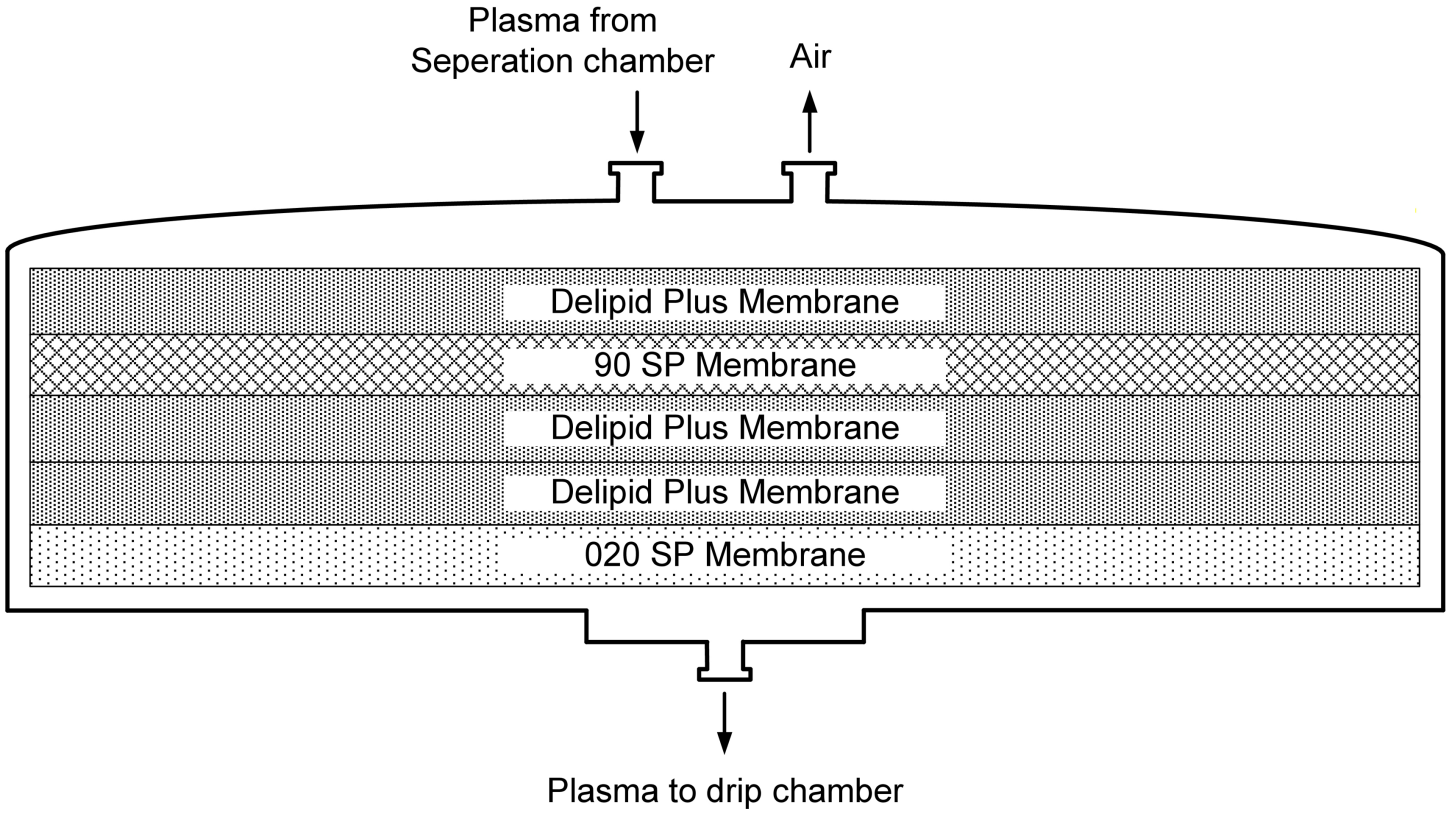
The structure of the JX-DELP filter.

**Figure 2:**
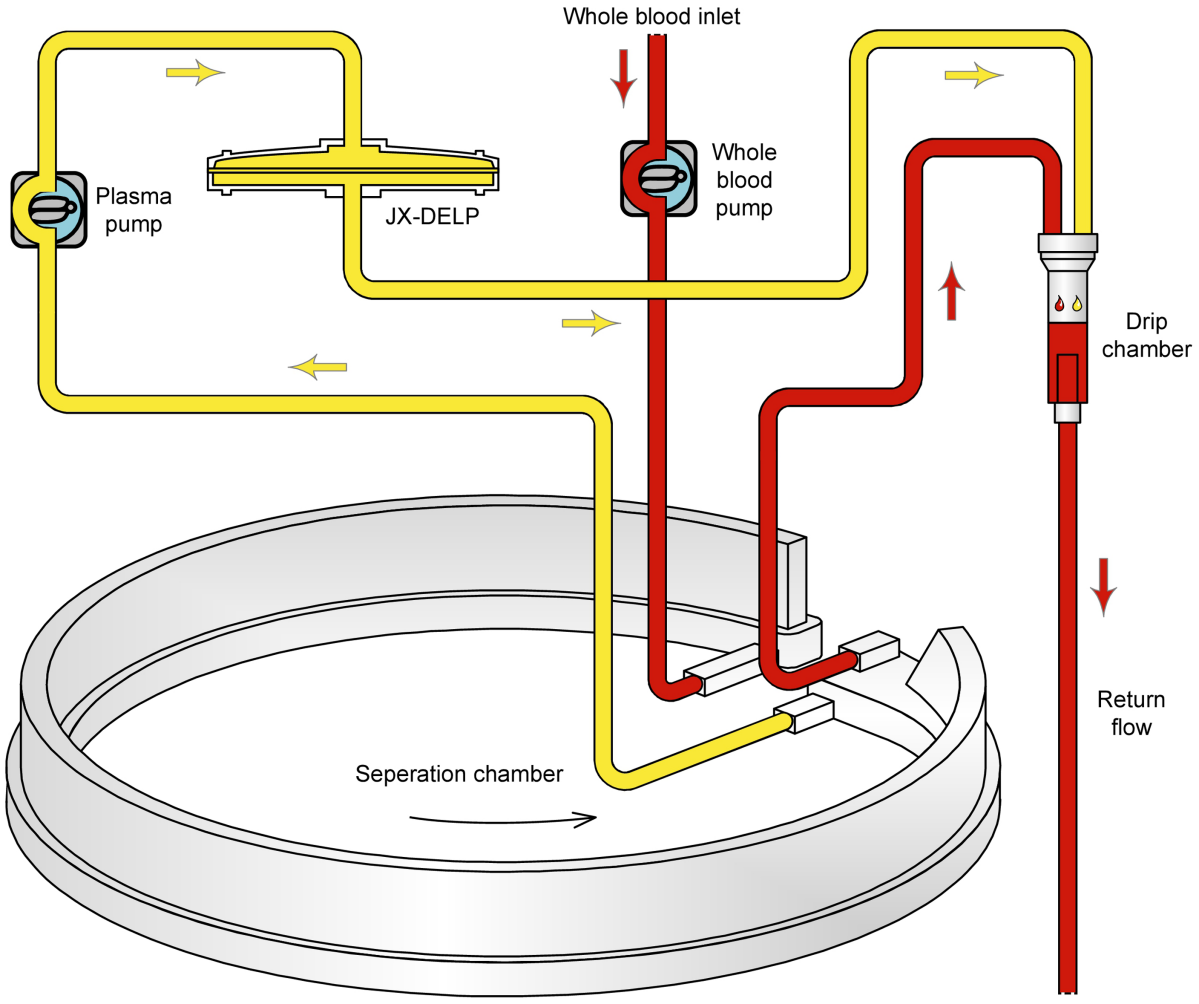
Disposable P1R plasma treatment set.

**Figure 3:**
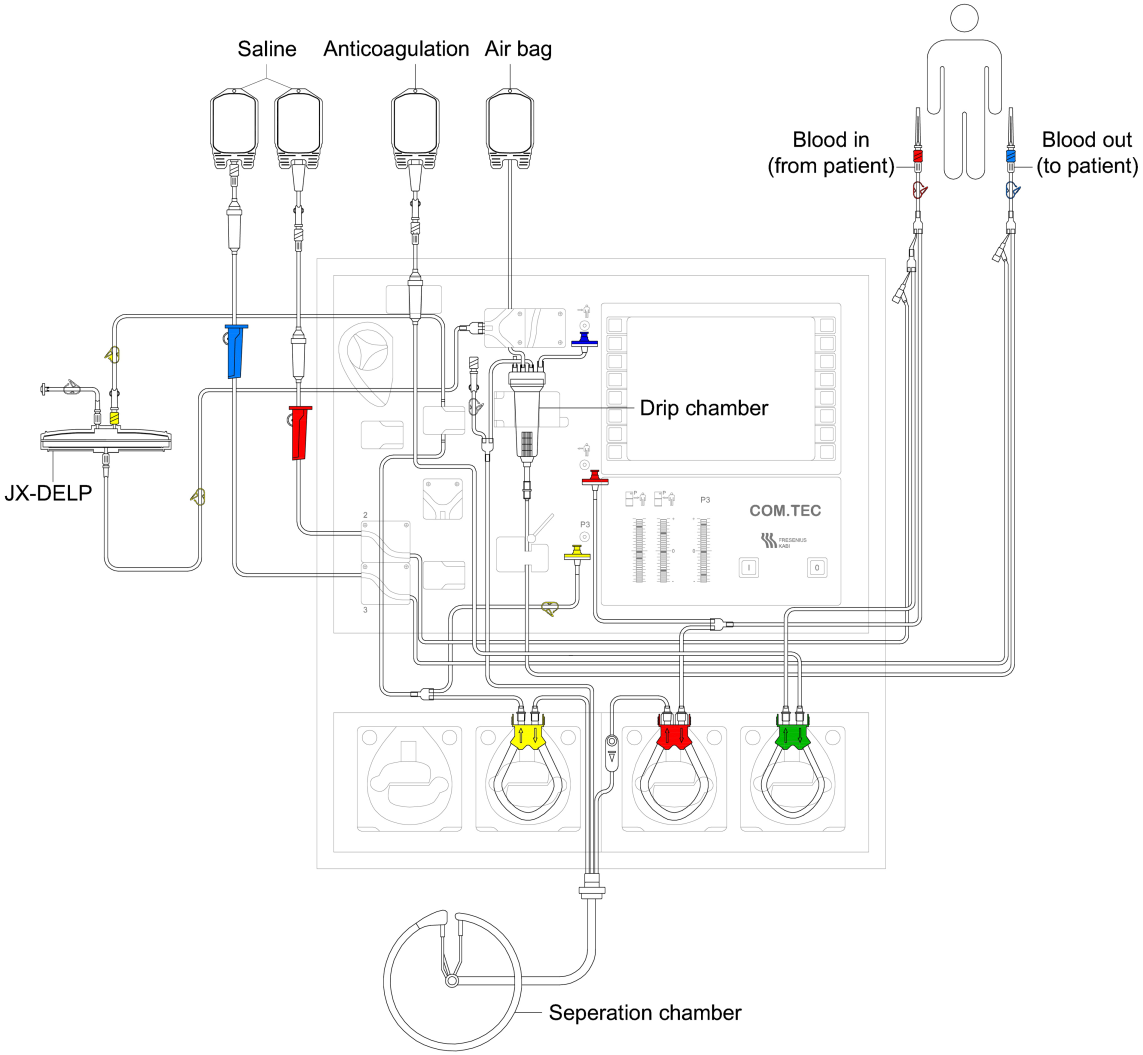
Schematic description of the separation process in the P1R separation chamber.

The DELP system is derived from an extracorporeal blood plasma lipid filtering method which has been patented in China (Patent No. ZL 2003 10108368.1) and the United States (Patent No. US 7,686,777 B2). The DELP system consists of an extracorporeal lipoprotein filter JX-DELP (Jiangxia Blood Technology Corporation, Shanghai, China), a COM.TEC cell separator and a P1R plasma treatment set (Fresenius HemoCare, Bad Homburg, Germany). The JX-DELP filter is composed of five layers of three cellulose-based filtration membranes (Delipid Plus membrane, 90SP membrane, and 020SP membrane) (Figure 1). The size of the JX-DELP filter is 215 mm (millimeter) in diameter and 35 mm in height. The COM.TEC cell separator was equipped with an innovative separation chamber for apheresis plasma. Centrifugation speed up to 2,200 revolutions per minute (rpm) with high force increased purity (Figure 2). The single use, disposable P1R plasma treatment set was applied throughout the treatment (Figure 3).

Prior to the treatment, the plasma filter was rinsed with 2000 mL of physiological saline. The DELP system procedure was performed via peripheral antecubital venous access or peripherally inserted central venous access at a blood flow rate of 20–50 mL/min. Sodium citrate was used as the anticoagulant (AC) at a ratio of 1:14 during the initial stage. During the treatment, the AC ratios were kept in the range of 1:10–1:16. 10% calcium gluconate infusion was required to prevent hypocalcemia related to the use of citrate anticoagulation. One standard DELP procedure was one third of the total plasma volume (800–1000 mL) processed.

The LDL-C treatment target for patients was <2.0 mmol/L or > 50% reduction in the present study according to AHA/ASA guidelines (2, 3).

### Vascular access for DELP system

Two separate venous access points in the extracorporeal circuit were established for the DELP system. Under normal conditions, peripheral antecubital venous access was established.

The large 20-gauge needle and the large 14-gauge needle were recommended for the blood outlet and inlet, respectively. For patients with poor peripheral venous access due to small veins or deep veins, peripherally inserted central venous access was required to maintain continuous blood flow. The 4F PowerPICC (Bard Medical, Inc., Covington, GA) was inserted into the antecubital vein using ultrasound-guided technique, which was routinely accessed for apheresis.

### Laboratory and safety investigations

For patients of the DELP group, fasting blood samples were collected on the day of DELP treatment and the next morning after treatment. Laboratory parameters, including plasma total cholesterol (TC), triglycerides, LDL-C, high-density lipoprotein cholesterol (HDL-C), blood cell counts, hemoglobin, prothrombin time (PT), activated partial thromboplastin time (APTT), fibrinogen (FIB), D-dimer, thrombin time (TT), total protein (TP), albumin, blood urea nitrogen (BUN), creatinine (CRE), blood potassium (K^+^), blood sodium (Na^+^) and blood calcium (Ca^2+^) were measured by routine methods in the laboratories of the Huashan Hospital. Blood pressure (BP) levels were recorded during the DELP process at six time points: baseline level before DELP treatment, 30, 60, 90 and 120 min after the initiation of blood flow through the device, and 2 h after DELP treatment. Adverse reactions were monitored during the whole period of the study. Any adverse events were documented.

### Neurological scores

For all patients, the National Institutes of Health Stroke Scale (NIHSS) scores (17) and the Modified Rankin Scale (mRS) scores (18) before treatment, 14 days and 90 days after stroke onset were assessed and recorded.

### Statistical analysis

All analyses were performed by statistical analysis software (Stata/SE 13.1). In the matched population, continuous variables were displayed as mean ± standard deviation (SD) or median and range. Most variables were tested statistically with Student’s *t*-test. Categorical variables were represented as numbers (n) and percentages (%) and were tested statistically with Fisher’s exact test. For all statistical analyses, *p* < 0.05 was considered statistically significant. Reduction rates were calculated according to the following formula,


$$\mathrm{Reduction}\left(\%\right)=\left(1-\frac{\mathrm{Con}c\mathrm{entration}\left[\mathrm{post}\right]}{\mathrm{Concentration}\left[pre\right]}\right)\times 100$$

## Results

We indentified 90 patients who underwent DELP during the study period. Pairwise matching created a final study cohort of 90 AIS patients with DELP and 90 controls.

For the DELP group, 138 DELP treatments were performed in 90 AIS patients (single treatment: *n* = 42, two treatments: *n* = 48). Demographic and clinical characteristics of the patients in the DELP group were summarized in Table 1. The mean age of patients including 70 males and 20 females was 61.73 ± 12.09 years. The average stroke onset time on admission was 2.5 ± 1.6 days. The average score of NIHSS before treatment was 6.91 ± 5.38. According to oxfordshire community stroke project (OCSP) classification, the partial anterior circulation stroke was the most common subtype in patients (58.89%). The background data of medication and anticoagulants were listed in Supplementary Table 1 and no significant difference were observed between two groups.

**Table 1:** Baseline characteristics of two groups.

	DELP group (*n* = 90)	Control group (*n* = 90)	*p* value
Male (n, %)	70 (77.78)	68 (75.56)	0.860
Age, years, Mean + SD	61.73 ± 12.09	63.04 ± 10.20	0.433
NIHSS, Mean + SD	6.91 ± 5.38	6.91 ± 5.46	1.000
LDL-C, mmol/L	3.15 ± 0.80	3.05 ± 0.71	0.384
rtPA thrombolysis (n, %)	16 (17.78)	19 (21.11)	0.707
OCSP (n, %)			0.933
Lacuna	7 (7.78)	6 (6.67)	
Partial anterior circulation	53 (58.89)	56 (62.22)	
Posterior circulation	24 (26.67)	21 (23.33)	
Total anterior circulation	6 (6.67)	7 (7.78)	

DELP, Delipid Extracorporeal Lipoprotein Filter from Plasma system; NIHSS, National Institute of Health Stroke Scale; rtPA, recombinant tissue-type plasminogen activator; OCSP, Oxfordshire Community Stroke Project.

To assess the safety of the DELP treatment, we analyzed laboratory parameters, blood pressure levels, and adverse event report forms for each patient. After a single DELP treatment, the average hematocrit (Hct) was reduced from 42.45 ± 4.32% to 41.02 ± 4.58% (*p* = 0.034), and no significant changes were observed in other hematological or biochemical laboratory parameters, including blood cell counts, coagulation parameters, albumin, creatinine, electrolytes, etc. In view of repetitive DELP treatments, significant reductions were only observed in Hct (from 41.87 ± 4.39% to 39.74 ± 4.84%, *p* = 0.026) and plasma total protein (from 69.92 ± 7.18 g/L to 65.21 ± 7.36 g/L, *p* = 0.002) (Table 2).

**Table 2:** Changes in blood lipid and routine laboratory parameters in single and repetitive DELP treatments.

Parameters (RR)	Single DELP treatment (*n* = 90) #			Twice DELP treatments (*n* = 48) #		
	Before therapy	After therapy	*p* value	Before 1st DELP	After 2nd DELP	*p* value
TC (2.8 ~ 5.9 mmol/L)	4.99 ± 1.01	3.56 ± 0.86	<0.001***	5.43 ± 1.03	3.16 ± 0.67	<0.001***
LDL-C (1.3 ~ 3.7 mmol/L)	3.15 ± 0.80	2.18 ± 0.63	<0.001***	3.42 ± 0.87	1.87 ± 0.48	<0.001***
HDL-C (0.8 ~ 1.8 mmol/L)	0.97 ± 0.26	0.92 ± 0.34	0.275	0.91 ± 0.23	0.87 ± 0.44	0.578
TG (<1.8 mmol/L)	2.01 ± 1.48	1.60 ± 1.03	0.033*	2.41 ± 1.83	1.48 ± 0.79	0.002**
WBC (3.5 ~ 9.5 × 109/L)	7.88 ± 2.37	8.03 ± 2.67	0.694	7.92 ± 2.27	7.52 ± 2.27	0.397
RBC (4.3 ~ 5.8 × 1012/L)	4.72 ± 0.61	4.57 ± 0.62	0.120	4.68 ± 0.62	4.42 ± 0.67	0.050
HGB (130 ~ 175 g/L)	144.27 ± 16.20	139.97 ± 16.23	0.077	142.06 ± 16.15	136.08 ± 15.45	0.067
HCT (40 ~ 50%)	42.45 ± 4.32	41.02 ± 4.58	0.034*	41.87 ± 4.39	39.74 ± 4.84	0.026*
PLT (125 ~ 350 × 109/L)	220.96 ± 52.18	209.40 ± 52.23	0.139	224.90 ± 60.20	218.08 ± 63.24	0.590
PT (10.4 ~ 12.9 s)	11.77 ± 2.70	12.66 ± 7.60	0.297	11.94 ± 3.59	11.95 ± 3.51	0.993
APTT (20.3 ~ 32.3 s)	23.53 ± 3.38	24.35 ± 4.31	0.157	22.93 ± 3.29	24.35 ± 4.01	0.061
FIB (1.8 ~ 3.5 g/L)	3.21 ± 1.17	2.96 ± 1.04	0.133	3.14 ± 1.35	2.93 ± 1.07	0.405
D-D (≤0.55FEUmg/L)	0.75 ± 1.21	0.60 ± 0.73	0.313	0.93 ± 1.53	0.66 ± 0.78	0.290
TT (14.0 ~ 21.0 s)	18.55 ± 2.76	18.68 ± 3.87	0.799	18.75 ± 3.02	18.24 ± 3.12	0.416
TP (65 ~ 85 g/L)	69.90 ± 6.98	68.16 ± 6.27	0.080	69.92 ± 7.18	65.21 ± 7.36	0.002**
ALB (35 ~ 55 g/L)	42.00 ± 3.91	40.91 ± 4.45	0.083	42.00 ± 4.01	40.40 ± 4.35	0.063
BUN (2.5 ~ 7.0 mmol/L)	5.63 ± 1.85	5.26 ± 1.70	0.160	5.73 ± 2.08	5.07 ± 1.96	0.114
CRE (50 ~ 130 μmol/L)	67.90 ± 17.84	66.59 ± 15.55	0.600	66.25 ± 19.33	66.02 ± 14.38	0.948
K^+^ (3.5 ~ 5.3 mmol/L)	3.78 ± 0.45	3.74 ± 0.41	0.522	3.89 ± 0.48	3.83 ± 0.33	0.470
Na^+^ (137 ~ 147 mmol/L)	141.11 ± 2.70	141.07 ± 2.46	0.908	140.46 ± 2.90	141.06 ± 2.45	0.274
Ca^2+^ (2.1 ~ 2.6 mmol/L)	2.20 ± 0.10	2.18 ± 0.10	0.261	2.22 ± 0.11	2.19 ± 0.11	0.130

#Results are shown as mean ± SD; DELP, Delipid Extracorporeal Lipoprotein Filter from Plasma system; RR, reference range; TC, total cholesterol; LDL-C, low-density lipoprotein cholesterol; HDL-C, high-density lipoprotein cholesterol; TG, triglycerides; WBC, white blood cell count; RBC, red blood cell count; HGB, hemoglobin; HCT, hematocrit; PLT, platelets; PT, prothrombin time; APTT, activated partial thromboplastin time; FIB, fibrinogen; D-D, D-dimer; TT, thrombin time; TP, total protein; ALB, albumin; BUN, blood urea nitrogen; CRE, creatinine; K+, kalium; Na+, sodium; Ca2+, calcium; *p < 0.05, **p < 0.01, ***p < 0.001.

The average blood pressure values at six time points during the 138 DELP treatments were shown in Supplementary Table 2. No significant fluctuations were observed in blood pressure levels. A single DELP treatment lasted less than 120 min. In the total 138 treatments, only two cases of lip numbness were reported during DELP treatment. The lip numbness was considered to be caused by low ionized calcium, and patients’ symptoms resolved after decreasing the flow rate of sodium citrate solution. No major or life-threatening adverse events were observed during the treatments. All 138 DELP treatments were successfully completed.

The effect on lipid concentrations of single DELP treatmenta were summarized in Table 2. LDL-C was significantly reduced from 3.15 ± 0.80 mmol/L to 2.18 ± 0.63 mmol/L (*p* < 0.001). The average concentration of TC was significantly decreased from 4.99 ± 1.01 mmol/L to 3.56 ± 0.86 mmol/L (*p* < 0.001) during single apheresis. Triglyceride was significantly decreased from 2.01 ± 1.48 mmol/L to 1.60 ± 1.03 mmol/L (*p* = 0.033). No significant decrease was observed in HDL-C after single DELP treatments (from 0.97 ± 0.26 mmol/L to 0.92 ± 0.34 mmol/L, *p* = 0.275).

The lipid-lowering effect of the DELP system was further confirmed in repetitive treatments (Table 2). The average removal rate of LDL-C was 45.32% (from 3.42 ± 0.87 mmol/L to 1.87 ± 0.48 mmol/L, *p* < 0.001). The level of TC was reduced by 41.80% (from 5.43 ± 1.03 mmol/L to 3.16 ± 0.67 mmol/L, *p* < 0.001). The removal rate of triglycerides was 38.59% (from 2.41 ± 1.83 mmol/L to 1.48 ± 0.79 mmol/L, *p* = 0.002). HDL-C was reduced from 0.91 ± 0.23 mmol/L to 0.87 ± 0.44 mmol/L (*p* = 0.578) after two DELP treatments.

Neurological scores were used to evaluate the influence of DELP treatments on the prognosis of AIS patients. The DELP and the control groups were comparable in most baseline parameters, including sex, age, NIHSS scores before treatment, percentage of rtPA intravenous thrombolysis, OCSP classification, LDL-C levels before treatment (Table 1). The neurological follow-up results showed that the NIHSS scores of the DELP group on the 14th (4.07 ± 3.77) and 90th (1.76 ± 1.96) day were significantly lower than those of the control group (14 days from onset: 5.36 ± 4.65, *p* = 0.043; 90 days from onset: 2.57 ± 2.90, *p* = 0.029) (Table 3). In addition, the DELP group had a higher ratio of mRS 0 to 1 (66.67%) than the control group (45.56%, *p* = 0.007) 90 days after stroke diagnosis (Table 3).

**Table 3:** Comparison of NIHSS and mRS scores between two groups.

	DELP group (*n* = 90)	Control group (*n* = 90)	*p* value
NIHSS, Mean + SD			
14th days	4.07 ± 3.77	5.36 ± 4.65	0.043*
90th days	1.76 ± 1.96	2.57 ± 2.90	0.029*
90th mRS (n, %)			0.019*
0	34 (37.78)	22 (24.44)	
1	26 (28.89)	19 (21.11)	
2	18 (20.00)	18 (20.00)	
3	9 (10.00)	17 (18.89)	
4	3 (3.33)	12 (13.33)	
5	0	2 (2.22)	

DELP, Delipid Extracorporeal Lipoprotein Filter from Plasma system; NIHSS, National Institute of Health Stroke Scale; mRS, modified Rankin Scale; *p < 0.05.

## Discussion

Since 1970s, several kinds of lipoprotein apheresis methods have been developed, including plasma exchange, double membrane filtration, dextran sulfate-coated cellulose beads, heparin-induced LDL precipitation, LDL hemoperfusion, etc. They mainly reduce blood lipids based on molecular weight, filter pore size, physisorption and immunoadsorption mechanisms (19, 20). The DELP system is a new intensive lipid lowering method based upon depth filtration materials and is approved for the treatment of AIS by the China Food and Drug Administration. Plasma filter of DELP system consists of five layers of cellulose-based filtration membranes, which can effectively absorb lipids through silicon dioxide nanoparticles with hydrophobic groups on the surface, and intercept molecules above 1300 kD or over 0.2 μm. Additionally, the advantages of DELP system mainly include low blood flow during the extracorporeal circuit, easier available access to blood flow, relatively low price and potential pleiotropic effects, which will be discussed below.

Using the DELP system, LDL-C was reduced by 30.79% with about 2000 mL of blood volume processed, and by 45.32% with 4000 mL of blood volume processed, respectively. Indeed, the DELP system could actually decrease the concentration of plasma LDL-C to the target level. Additionally, TC and triglycerides were also reduced significantly by a single DELP treatment. HDL-C was reduced by 5.15% in single DELP treatment and 4.40% in two DELP treatments, respectively. The reduction rate was close to other reports concerning lipoprotein apheresis (10, 12, 21–23), and was insufficient for statistical significance.

In view of safety, firstly, only Hct and total protein were decreased significantly during DELP treatments. Such changes have been reported in some lipoprotein apheresis systems before, and those reductions could usually fully recover in a few days after apheresis (12, 21). No other significant changes were observed in hematological or biochemical parameters after single or repetitive DELP treatments, which may be attributed to the simple compositions and small quantity of rinsing solution required for DELP treatments.

Secondly, low blood flow in DELP treatment may help to prevent hypovolemia and hypotension. Blood pressure lowering may reduce cerebral perfusion which is associated with severe stroke events (24, 25). The required flow (20–40 mL/min) for the DELP system was lower than in other lipoprotein apheresis systems (10, 22). The COM.TEC device was applied for plasma collection, and the maximum extracorporeal blood volumes could be decreased to 126 mL. Owing to low blood flow and extracorporeal blood volumes, all patients including those with posterior circulation stroke and low body weight index completed DELP treatments and their blood pressure remained stable during the extracorporeal circuit. LDL-C lowering efficiency of different lipoprotein apheresis methods was summarized in Supplementary Table 3, which included 7 lipoprotein apheresis methods.

Moreover, access to blood flow was reliable, safe and satisfactory to patients in DELP treatments. We paid particular attention to vascular access management and needle selection. The needles were placed by puncture in superficial veins of the upper limb. Many AIS patients were admitted to the hospital with non-visible or non-palpable peripheral veins or disorders of consciousness, often resulting in multiple painful attempts at cannulation. Routinely, this would result in requiring a central venous catheter for the patients (26, 27). Ultrasound-guided peripherally inserted central venous access could be established for DELP. In a systematic review of six randomized control trials, the effect of ultrasound guidance versus traditional approaches to cannulation was investigated and the results suggested a fourfold increase in the success rates of ultrasound group (28). The ultrasound-guided PowerPICC catheter was an adequate venous access tool which could even support power injection of contrast media or blood sampling (29).

Additionally, the cost of a single DELP treatment, including JX-DELP plasma filter and tubing set was about $1700 (covered by National Healthcare Security Insurance). Above all, safety on extracorporeal circulation and operation feasibility made the DELP system applicable for intensive lipid lowering treatment for AIS patients.

Combined application of lipoprotein apheresis and lipid modifying agents may have complementary advantages. Lipoprotein apheresis could achieve a noticeable LDL-C reduction within hours via physical principle without worrying about medication side effect (30–32). Therefore, we hypothesized that combined therapy of lipid modifying drugs following initial lipoprotein apheresis may be a promising method for lipid management for AIS patients. More prospective trials are needed.

Furthermore, the DELP system may exert pleiotropic effects except for LDL-lowering capability in the treatment of ischemic stroke. Previous investigations indicated that lipoprotein apheresis may play a role in reducing plasma viscosity, improving endothelial function, anti-inflammatory and free radical scavenging (10, 11, 33–39).

Xue et al. found DELP treatment could decrease neuronal apoptosis in experimental stroke models (40). A retrospective study in venous thrombotic patients with ischemic stroke conducted by Cui et al. showed an increase in the proportion of mRS 0 ~ 1 at 90 days (*p* = 0.042) in the DELP group compared with NO DELP group (41). More prospective control trials are needed to evaluate the effect of the DELP treatment on the prognosis of AIS patients.

Elevated serum lipoprotein (a), also referred to as Lp (a), is a risk factor for atherosclerotic cardiovascular disease (ASCVD) (42). It can be removed by DELP system and need to be proved precisely in the future.

## Limitations

The present study had certain limitations that needed to be considered. Firstly, this was a relatively small-sample single-center retrospective study and no randomization was performed. Matching on factor is only age. Secondly, due to the particularity of the treatment, blinding method could not be set up. Thirdly, the effect of lipid modifying drugs may be considered a potential factor influencing plasma lipids. Nonfasting blood samples were not collected after DELP treatment immediately. However, all DELP treatments were completed within 72 h and the effect of lipid modifying drugs may be relatively small. Last but not least, we do not have enough data on the influence of DELP treatment in long-term follow-up. Another notable limitation is the absence of documented evidence regarding Lp(a) as a risk factor for atherosclerotic cardiovascular disease. These investigations should be undertaken in the future.

## Conclusion

This is the first clinical data on the operating protocol, LDL-C lowering capability and safety of DELP system. The present study confirmed the effectiveness and safety of DELP therapy for patients with AIS. Moreover, operation feasibility, low extracorporeal volume and low flow rate made the DELP system suitable for AIS patients. Thus, the DELP system may be a promising lipid-lowering treatment choice for AIS patients.

## Data availability statement

The original contributions presented in the study are included in the article/Supplementary material, further inquiries can be directed to the corresponding authors.

## Ethics statement

The studies involving humans were approved by Ethics Committee of Huashan Hospital, Fudan University. The studies were conducted in accordance with the local legislation and institutional requirements. The participants provided their written informed consent to participate in this study. Written informed consent was obtained from the individual(s) for the publication of any potentially identifiable images or data included in this article.

## Author contributions

YJ: Writing – original draft, Writing – review & editing, Methodology, Formal analysis, Visualization. QY: Methodology, Writing – original draft, Writing – review & editing. TY: Writing – original draft, Writing – review & editing. JZ: Investigation, Writing – original draft. QL: Writing – review & editing. XH: Project administration, Writing – review & editing. QD: Project administration, Writing – review & editing.

## Supplementary material

SUPPLEMENTARY FIGURE 1 The flowchart of the study.
