# Supplementary material for: Delipid extracorporeal lipoprotein filter from plasma system: a new intensive lipid lowering therapy for patients with acute ischemic stroke
Source: Front Neurol. 2024 Mar 6;15:1342751. doi: 10.3389/fneur.2024.1342751 (PMC10950928; doi:10.3389/fneur.2024.1342751)
Supplement: Supplementary file 1 [file Data_Sheet_1.pdf]

SUPPLEMENTAL TABLE 1 Cerebral risk factors and antiplatelet therapy of two groups

	DELP group （n=90）	Control group （n=90）	*P* value
Male(n,%)	70 (77.78)	68 (75.56)	0.860
Age，years, Mean+SD	61.73±12.09	63.04±10.20	0.433
Vascular risk factors (n, %)			
Hypertension	34 (37.8)	39 (43.3)	0.544
NIDDM	28 (31.1)	23 (25.6)	0.508
Current smoker	21 (23.3)	18 (20.0)	0.718
Obesity	34 (37.8)	28 (31.1)	0.433
Antiplatelet therapy (n, %)			0.751
Mono antiplatelet therapy	59 (65.6)	62 (68.9)	
Dual antiplatelet therapy	31 (34.4)	28 (31.1)	

DELP, Delipid Extracorporeal Lipoprotein Filter from Plasma system; NIDDM, non-insulin-dependent diabetes mellitus;

* P<0.05，**P<0.01，*** P<0.001.

SUPPLEMENTAL TABLE 2 Influence on blood pressure of single DELP treatment (n=138 times)

	Baseline	30min	60min	90min	120min	2h after treatment	*P* value
SBP, mmHg, mean±SD	142.4±21.3	142.7±22.0	141.8±20.4	141.3±21.9	141.6±21.9	139.7±22.6	0.906
DBP, mmHg, mean±SD	78.8±9.6	78.3±9.0	77.9±8.5	79.1±8.9	78.0±8.8	78.7±8.5	0.758

SBP, systolic blood pressure; DBP, diastolic blood pressure

SUPPLEMENTAL TABLE 3 LDL-C lowering efficiency of different lipoprotein apheresis methods

Lipoprotein apheresis method	Number of patients	Mean treatment interval	Treatment times	Reduction rate of LDL-C level	Treatment volume of a single session	Flow rate (ml/min)	Duration of a single treatment	References
DELP	48	Two treatments within 3 days	2	45.3 %	800~1000 ml plasma	20~50	<120 min	This study
HELP	48	Two treatments within 8 days	2	45.8%	2500~3000 ml plasma	60~80	2~3 hours	Walzl, M. , et al. Stroke. 1993 24.10:1447-51.
HELP	91	Two treatments within 8 days	2	51.8%	2500~3000 ml plasma	60~80	2~3 hours	Walzl, M. , et al. Angiology, 1997 48.12:1031-1036.
IMAL	8	Weekly	4	81.6%	6000 ± 0 ml plasma	50~80	195 ± 20 min	Schmaldienst, S , et al.
DSA	8	Weekly	4	84.6%	5090 ± 600 ml plasma	50~80	187 ± 29 min	Atherosclerosis, 2000
DALI	8	Weekly	4	74.6%	7171 ± 1329 ml whole blood	50~80	135 ± 20 min	151.2:493-499.
Liposorber D	10	Weekly or biweekly	7~11	62.2 ± 11.5 %	8218 ± 1472 ml whole blood	60~100	112 ± 22 min	Carsten, et al. Artificial Organs, 2003 27(12):1116-1122.
KLD01	33	Once every 2 weeks	5	61.5 ± 6.2 %	9200 ± 1200 ml whole blood	NA	138 ± 20 min	Hiromi Tasaki, et al. Therapeutic Apheresis & Dialysis, 2006 10(1):32-41
DALI	14	Weekly	17	64~76 %	7675 ml whole blood	60	2 hours	Dräger, et al. European Journal of Clinical Investigation, 2015 28.12:994-1002.
