# Supplementary figures and images for: Delipid extracorporeal lipoprotein filter from plasma system: a new intensive lipid lowering therapy for patients with acute ischemic stroke
Source: Front Neurol. 2024 Mar 6;15:1342751. doi: 10.3389/fneur.2024.1342751 (PMC10950928; doi:10.3389/fneur.2024.1342751)

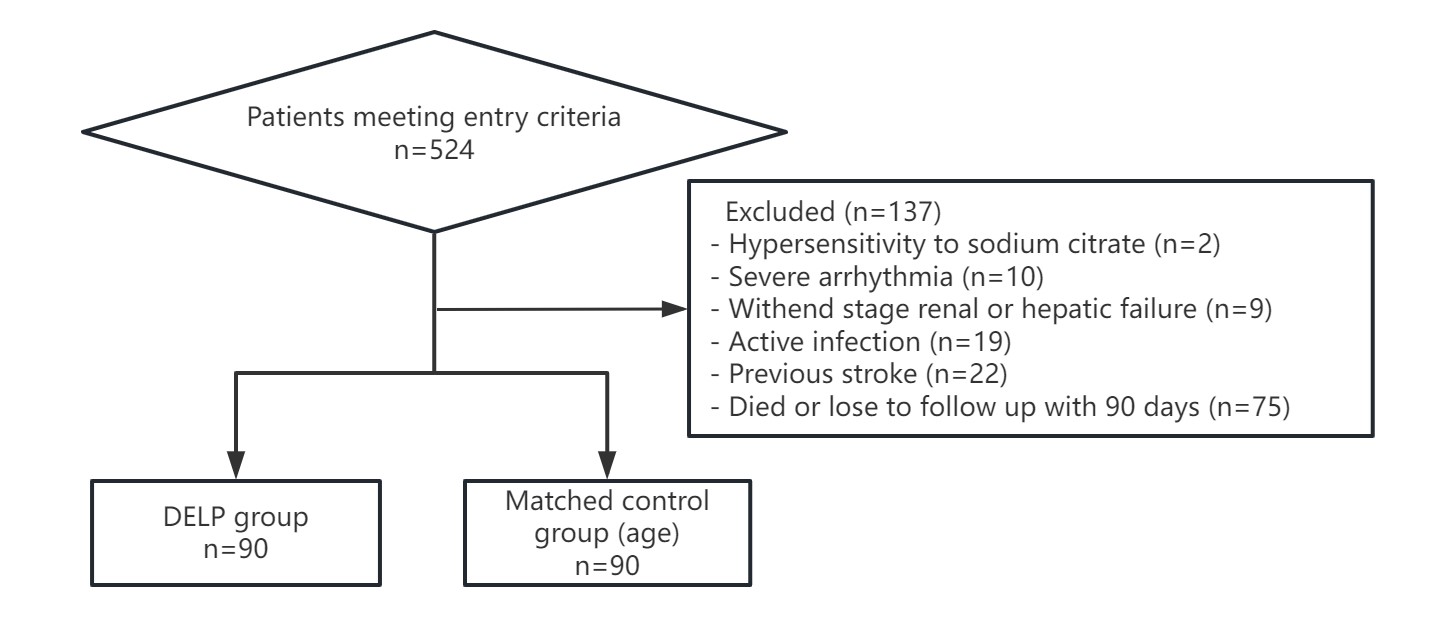

Supplement: SUPPLEMENTARY FIGURE 1 — The flowchart of the study. [file Image_1.jpg]
